# Clinical validation of optimised RT-LAMP for the diagnosis of SARS-CoV-2 infection

**DOI:** 10.1038/s41598-021-95607-1

**Published:** 2021-08-10

**Authors:** Boon Lim, Jeremy Ratcliff, Dorota A. Nawrot, Yejiong Yu, Harshmeena R. Sanghani, Chia-Chen Hsu, Leon Peto, Simon Evans, Susanne H. Hodgson, Aikaterini Skeva, Maria Adam, Maria Panopoulou, Christos E. Zois, Katy Poncin, Sridhar R. Vasudevan, Siqi Dai, Shuai Ren, Hong Chang, Zhanfeng Cui, Peter Simmonds, Wei E. Huang, Monique I. Andersson

**Affiliations:** 1 Department of Engineering Science, University of Oxford, Parks Road, Oxford, OX1 3PJ UK; 2 Department of Engineering Science, Institute of Biomedical Engineering, University of Oxford, Oxford, OX3 7DQ UK; 3 Peter Medawar Building for Pathogen Research, Nuffield Department of Medicine, University of Oxford, Oxford, OX1 3SY UK; 4 Department of Pharmacology, University of Oxford, Mansfield Road, Oxford, UK; 5 Department of Microbiology, Oxford University NHS Foundation Trust, Oxford, UK; 6 Jenner Institute, Nuffield Department of Medicine, University of Oxford, Oxford, UK; 7 Democritus University of Thrace, University Hospital of Alexandroupolis, Alexandroupoli, Greece; 8 Sir William Dunn School of Pathology, University of Oxford, Oxford, UK; 9 Oxford Suzhou Centre for Advanced Research (OSCAR), University of Oxford, Suzhou, China; 10 Nuffield Division of Clinical Laboratory Science, Radcliffe Department of Medicine, University of Oxford, Oxford, UK

**Keywords:** Clinical microbiology, Molecular medicine

## Abstract

We have optimised a reverse transcription-loop-mediated isothermal amplification (RT-LAMP) assay for the detection of SARS-CoV-2 from extracted RNA for clinical application. We improved the stability and reliability of the RT-LAMP assay by the addition of a temperature-dependent switch oligonucleotide to reduce self- or off-target amplification. We then developed freeze-dried master mix for single step RT-LAMP reaction, simplifying the operation for end users and improving long-term storage and transportation. The assay can detect as low as 13 copies of SARS-CoV2 RNA per reaction (25-μL). Cross reactivity with other human coronaviruses was not observed. We have applied the new RT-LAMP assay for testing clinical extracted RNA samples extracted from swabs of 72 patients in the UK and 126 samples from Greece and demonstrated the overall sensitivity of 90.2% (95% CI 83.8–94.7%) and specificity of 92.4% (95% CI 83.2–97.5%). Among 115 positive samples which Ct values were less than 34, the RT-LAMP assay was able to detect 110 of them with 95.6% sensitivity. The specificity was 100% when RNA elution used RNase-free water. The outcome of RT-LAMP can be reported by both colorimetric detection and quantifiable fluorescent reading. Objective measures with a digitized reading data flow would allow for the sharing of results for local or national surveillance.

## Introduction

The rapid spread of SARS-CoV-2 has challenged the testing capacity of many countries. In the United Kingdom, the testing strategy early on in the outbreak prioritized identifying and protecting essential workers and vulnerable patients and, during the height of the outbreak, only those who were symptomatic were tested^1^. However, to effectively control localized outbreaks as governments begin to ease lockdown policies, a wider testing strategy achieved by decentralised and rapid testing capacity will be of the utmost importance. Currently, reverse transcription, real-time quantitative polymerase chain reaction (RT-qPCR) detection of SARS-CoV-2 RNA remains the gold standard for the diagnosis of COVID-19^2^. Whilst largely reliable and able to evaluate samples through high-throughput processes, RT-qPCR requires trained personnel, RNA extraction, and sophisticated instrumentation, all of which limit the use of RT-qPCR test for decentralized testing. In addition, the rapid rise in the global demand for RT-qPCR testing has resulted in a global shortage in necessary supplies^3^.

We propose a reverse transcription, loop mediated isothermal amplification (RT-LAMP) assay for the detection of SARS-CoV-2 RNA. The RT-LAMP assay is a colorimetric nucleic acid amplification assay—successful amplification of the target sequence results in a colour change from pink to yellow. The reaction takes place in a single-tube format and simply requires heating at 65 °C for 30 min for the reaction to proceed. The RT-LAMP assay is rapid, single-step, and ideal for point-of-care-testing (POCT) as the only equipment required is a heating platform. The assay endpoint can be assessed using a variety of outputs including colour change, fluorescence and absorbance^4^. By shortening the sample collection, testing, and readout, the POCT RT-LAMP assay is particularly suited to settings where real-time results would directly impact on patient care, including mobile testing centres, emergency departments, primary care facilities, residential homes, and airports^5^.

Several groups have reported RT-LAMP assays for detecting SARS-CoV-2 RNA^6–10^. Our RT-LAMP assay method has been previously presented^11^. There, we demonstrated the ability of our O117 primer set to reliably detect 20 copies per reaction of an Orf1ab RNA transcript. In this project, we have extended on our previous formulation in several ways to further streamline and stabilise the performance of this assay as a POCT outside of standard diagnostic laboratories. We validate the new formulation using several commercial SARS-CoV-2 transcripts, residual oropharyngeal clinical samples and saliva clinical samples.

## Materials and methods

### Primer design

Primers were designed as previously described in Huang et al.^11^. The primer set O117 targeting Orf1ab of SARS-CoV-2 was designed using PrimerExplorer (http://primerexplorer.jp/e/)^12^ and each primer was synthesised by Integrated DNA Technologies (IDT, UK). The Switch was synthesised by Integrated DNA Technologies (IDT, UK) with an Iowa Black Dark Quencher at the 3′ end (See Supplementary Table S1).

10× O117_N primer mix was prepared by mixing equal volumes of 16 μM FIP, 16 μM BIP, 2 μM F3, 2 μM B3, 4 μM LF and 4 μM LB.

10× O117_Q primer mix was prepared by mixing equal volumes of 16 μM FIP, 16 μM BIP, 2 μM F3, 2 μM B3, 4 μM LF, 4 μM LB and 24 μM Switch.

### RT-LAMP reaction mix preparation and generalized procedure

All equipment, laminar-flow cabinets, and working benches were sprayed with 70% w/v ethanol (Sigma-Aldrich Co., UK) and RNaseZap (Sigma-Aldrich Co. UK) prior to reaction mix preparation. All reagents and PCR tubes were kept on ice during kit preparation.

For wet reaction mix, 12.5 μL of WarmStart Colorimetric LAMP 2× Master Mix (DNA & RNA) from New England Biolabs (New England Biolabs, UK), 5 μL of DNase & RNase-free molecular grade water (Thermo Fisher Scientific Ltd), and 2.5 μL of 10× O117_N or 10× O117_Q primer mix was added into PCR tubes and mixed homogeneously.

For dried reaction mix, 12.5 µL 2× Master Mix was supplemented with 7.5 µL drying protective agent, containing sucrose, dextran, lactose and trehalose. 2.5 µL of 10× O117_N or 10× O117_Q primer mix was then added into the PCR tubes. The PCR tubes with caps open were dried with a freeze dryer (VirTis Genesis Pilot Lyophilizer, SP Scientific). After desiccated, importing nitrogen gas to release the vacuum. Seal the caps of the PCR tubes under nitrogen environment. The dried reaction mixes were stored at − 20 °C immediately.

The RT-LAMP reaction was run by adding 5 μL of sample of interest to a 20 μL wet reaction mix or a dried reaction mix resuspended in 20 μL of DNase/RNase-free water and heating the reaction at 65 °C for 30 min. A positive result was confirmed through a pH-dependent colorimetric change or confirmation of the production of LAMP product using absorbance or fluorescence.

### Full-length transcripts

Three full-length SARS-CoV-2 RNA transcripts were used in this study. Research reagent 19/304 (NIBSC, UK) was the kind gift of Giada Mattiuzo. Synthetic RNA Control 1—MT007544.1 (Twist Bioscience Ltd) and Synthetic RNA Control 2—MN908947.3 (Twist Bioscience Ltd) were the kind gifts of John Taylor (Oxford Medical Genetics Laboratories).

### Clinical samples

This study is to evaluate the optimised RT-LAMP by comparing it with conventional RT-qPCR. This is not clinical trial and this study is not used as clinical diagnosis. We confirm that that all methods were carried out in accordance with relevant guidelines and regulations. All experimental protocols were approved by the Joint Research Office study classification group in Oxford University. The informed consent was obtained from all subjects or, if subjects are under 18, from a parent and/or legal guardian. Residual samples from clinical testing of 72 symptomatic patients from the Oxford University Hospitals NHS Foundation Trust, Department of Microbiology, were accessed for the evaluation. This study did not require formal ethical approval as is classified as a service evaluation. The study was registered and accepted as a service evaluation on Oxford University Hospitals Governance System Ulysses register (CSS-MICRO-6330). The 126 clinical samples from University Hospital of Alexandroupolis in Greece. The study protocol was approved by the local committee of ethics and deontology in accordance with the Declaration of Helsinki (Number 44/30-7-2020). All samples have informed consent to be used for research and validation studies on an anonymous basis after deidentification.

#### RT-qPCR

Briefly, for the patient samples oro-nasopharyngeal swabs were stored in Universal Transfer Medium (COPAN Diagnostic, USA) or 0.9% saline and extracted using the QIAsymphony system (Qiagen Co., UK). 200 µL of sample solution was mixed with 430 µL of Buffer OBL and carried into the extraction phase. RNA was eluted into 60 µL of buffer AVE and 10 µL of the elution was carried into a RT-qPCR amplification using the Rotor-Gene Q (Qiagen Co., UK). RT-qPCR reactions were set up using a RNA polymerase gene target validated by Public Health England (PHE, UK) and the Altona RealStar SARS-CoV-2 RT-PCR Kit targeting the E and S genes.

The deep-throat saliva samples were taken from 527 participants and diluted with PBS in 1:1 ratio. Diluted DTS of 200 µL was added to the binding buffer of MagMAX Viral/Pathogen Nucleic Acid Isolation Kit (Thermo Fisher Scientific) and viral RNA extraction was carried out according to the manufacturer protocol with the exception that RNA was eluted in 60 µL RNase-free water instead of the elution buffer. The entire isolation process was done in Kingfisher Flex automated system (Thermo Fisher Scientific). RT-PCR for detecting extracted RNA of SARS CoV-2 was carried out according to the US CDC 2019-Novel Coronavirus (2019-nCoV) Real-Time RT-PCR Diagnostic Panel Instructions for Use. RT-PCR targeted N1, N2 of SARS-CoV-2 genes and human RNaseP gene. Briefly, 5 µL of extracted RNA or control, 1.5 µL combined primer/probe mix (IDT, US), 5 µL 4× TaqMan Fast Virus 1-Step Master Mix (Thermo Fisher Scientific) and 8.5 µL RNase-free water were mixed (total volume 20 µL) and loaded into real-time PCR system (Applied Biosciences ViiA7, Thermo Fisher Scientific). The data were analysed using the QuartStudio Real-Time PCR software (Thermo Fisher Scientific).

#### RT-LAMP

RT-LAMP for detecting SARS CoV-2 was carried out according to the previous description^11^. For the wet reaction mix, 5 μL of the eluted RNA sample was carried into a reaction containing O117_Q for RT-LAMP assay. For dry reactions, 5 µL of extracted RNA was mixed with 20 µL RNase-free water to reconstitute lyophilized RT-LAMP master mix. The reaction was incubated at 65 °C for 30 min and the colour was recorded.

### Analytic stability, sensitivity, and specificity

The stability of the wet reaction mix containing O117_N or O117_Q was assessed. 5 μL of 40 copies/µL of Synthetic RNA Control 2 was added as a positive control and 5 μL of 0.2 μg/µL human genome cDNA was added as a negative control. Human genome cDNA was reverse transcribed from whole RNA extracted from human bone marrow derived MSC line (Lonza, UK). RNA extraction was performed as previously described^13,14^ and reverse transcribed using the QuantiTect Reverse Transcription kit (Qiagen, Manchester, UK). 0.5–1 μg whole RNA was converted into cDNA per manufacturer’s instructions including a genomic DNA wipe-out step. Final cDNA sample was diluted to 0.2 μg/μL in RNAse/DNAse free water and stored at − 20 °C until used for RT-LAMP assay as negative control. The reaction was run as described above and the colour change was assessed at 0, 30, and 60 min.

To obtain an estimate of analytic sensitivity, the 50% endpoint of the LAMP assay was assessed using the three full-length SARS-CoV-2 transcripts. 19/304 was extracted using the viral RNA mini kit (Qiagen Co., UK) and eluted in buffer AVE. Serial dilutions of 5 µL of RNA in buffer AVE were used to spike 20 µL of solution from a 50 µL negative throat swab resuspended in 450 µL of RNase-free water. 25 µL of solution was added to a dried kit containing O117_N or O117_Q primers and were tested in parallel and with five replicates. The LAMP assay was run as described above and assessed via colour change and confirmed via UV–Vis of LAMP product on a 2% agarose gel stained with Sybr-Safe (Thermo Fisher Scientific). The 50% endpoint was calculated using the Reed-Muench method^15^.

The specificity of the LAMP assay for SARS-CoV-2 RNA against other human-infective coronaviruses was assessed. RNA was extracted from biobanked respiratory samples positive for OC43, HKU1, NL63, and 229E (matched samples were previously collected and processed as described in Gaunt et al.^16^) using the QIAamp Viral RNA Mini Kit (Qiagen Co., UK) according to the manufacturer’s instructions. 20 µL of solution from a 50 µL negative throat swab resuspended in 450 µL of RNase-free water was spiked with 5 µL of the RNA eluate. 25 µL was added to a dried kit containing O117_N or O117_Q primers and were tested in parallel. The LAMP assay was run in duplicate at 65 °C for 40 min and assessed via colour change.

### Quantitative detection methods

Three methods for converting the qualitative colour change output of RT-LAMP reactions into a quantitative output were assessed. The absorbance spectrum of negative and positive samples was read using a Fluorostar Omega microplate reader (BMG Labtech, UK), and the reaction outcome was assessed using the ratio of 430/560 nm. Detection of the LAMP product was also performed using an intercalating fluorescent dye, SYTO 9, at a final concentration of 3 µM (Thermo Fisher Scientific), added to the reaction mix once the 30 min RT-LAMP reaction was completed. Excitation and emission were performed at 485 nm and 500 nm, respectively by Fluorostar Omega microplate reader (BMG Labtech, UK). Alternatively, 5 µL of the reaction product was carried into the Qubit dsDNA BR Assay Kit (Invitrogen, UK). The assay was performed according to the manufacturer’s instructions and read using a Qubit 2.0 fluorometer. Cutoff for positive results for absorbance and SYTO 9 quantitative assays were determined to be three times the standard deviations above the negative controls. For Qubit, three times the standard deviation was calculated using negative samples and negative controls.

## Results

### Temperature dependent oligo switch stabilises RT-LAMP assay

Several reports have indicated that carryover contamination and off target amplification can result in false positives when conducting RT-LAMP assays^17–20^. To address the problem of self- and off-target amplification, we introduced a short oligonucleotide designated as a switch, whose sequence is complementary to one of the primers used in the LAMP reaction and 3′-end was modified by a dark quencher molecule—Iowa Black RQ (IBRQ). The switch serves as a temperature-dependent switch to bind an essential primer of LAMP (e.g. FIP), limiting non-specific amplification and the formation of primer dimers. A working hypothesis for the mechanism of action is that when the temperature is below the working temperature of LAMP (e.g. 65 °C), the FIP primer is bound to the switch oligonucleotide. The switch likely prevents non-specific amplification of non-SARS-CoV-2 RNA eluted during sample processing by binding the FIP primer with higher affinity than contaminating nucleic acids. At the working temperature, the switch competes with the sample RNA for binding, allowing FIP to bind target RNA/DNA for reverse transcription and amplification of target nucleic acids. The mechanism should be further defined with additional experimentation but that is out of the scope of this study.

**Figure 1:**
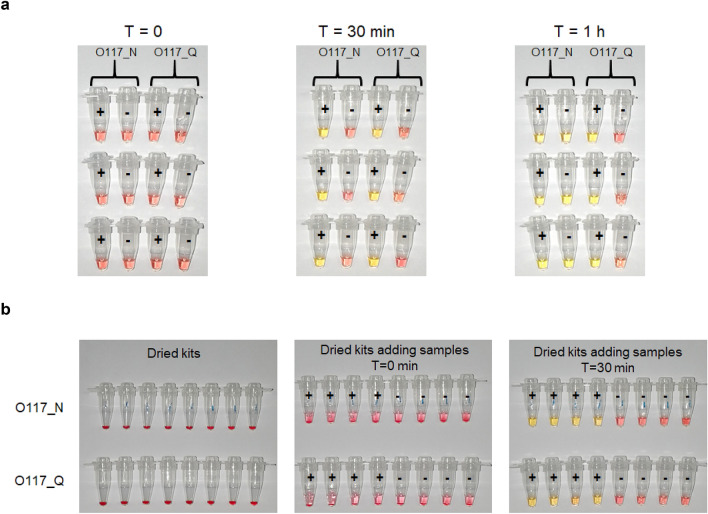
Stability test of improved RT-LAMP reaction mix. (**a**) Stability test of RT-LAMP reaction mix containing O117_N and O117_Q. Tubes were added with Synthetic RNA Control 2—MN908947.3 (‘+’) or human genome cDNA (‘−’) followed by heating at 65 °C for the time indicated by T. Each case has three replicates. (**b**) Dried reaction mixes stored at room temperature up to 3 days. Then they mixed with 20 µL DNase/RNase free water and 5 µL of Synthetic RNA Control 2—MN908947.3 (‘+’) or human genome cDNA (‘−’). The tubes were incubated at 65 °C for 30 min. Top row: Dried reaction mixes containing O117_N; Bottom row: Dried reaction mixes containing O117_Q.

The O117 primer set^11^ was used to evaluate the performance of the switch. Two preparations of the primer mix were used throughout this study: O117_N contains the original six LAMP primers, and O117_Q contains the six LAMP primers and a switch oligonucleotide (Table 1). The blank controls without target template have been previously shown to result in false positive due to non-template amplification^21^. In this study, a 12-bp switch with 3′-end modification with IBRQ (IDT, UK) was designed to bind FIP and block self or off-target amplification when the temperature is below the RT-LAMP working temperature. O117_N or O117_Q primers were used to test Synthetic RNA Control 2—MN908947.3 (Twist Bioscience, US) and human cDNA (Sigma-Aldrich UK) through a prolonged incubation of up to 1 h. WarmStart Colorimetric Master Mix (New England Biolabs, UK) was used for the RT-LAMP assay. After incubating at 65 °C for 30 min, both O117_N and O117_Q LAMP primers, showed positive (yellow) for 200 copies of Synthetic RNA Control 2, and negative (pink) for the human cDNA control (Fig. 1a). However, the O117_N primer mix turned yellow within 1 h of heating, representing a false positive, while the O117_Q mix maintained a pink colour throughout the experiment (Fig. 1a). We also showed that O117_N primer set could generate up to 60% false positive result, whilst O117_Q can perform reliably after incubating at 65 °C for one hour (Supplementary Fig. S1). The results suggest that a switch in O117 primers reduces off-target amplification at lower temperature and stabilises the LAMP assay at reaction temperature.

**Table 1:** Primer sequences used in O117_N and O117_Q primer mix.

O117 primer	Sequence (5′–3′)
FIP	GGTTTTCAAGCCAGATTCATTATGGATGTCACAATTCAGAAGTAGGA
BIP	TCTTCGTAAGGGTGGTCGCAGCACACTTGTTATGGCAAC
F3	CCCCAAAATGCTGTTGTT
B3	TAGCACGTGGAACCCAAT
LF	TCGGCAAGACTATGCTCAGG
LB	TTGCCTTTGGAGGCTGTGT
Switch	GGCTTGAAAACC-IBRQ

*IBRQ* Iowa Black Dark Quenchers.

### Freeze-drying improve reaction mix storage and transport

One limitation of the deployability of RT-LAMP to developing countries and rural areas is the need for cold-chain transport of reagents. To overcome this limitation, we freeze-dried a complete reagent mix, including Bst2.0 DNA polymerase, WarmStart reverse transcriptase, colorimetric indicator, dNTPs, primers, and buffer in one PCR tube to make a dry and ready-use reaction mixture. The stability of the dried reaction mix was assessed by storing at room temperature for 15 days before RT-LAMP assay was performed (Fig. 1b and Figs. S2 and S3). On day 3, the dried mixes were first re-suspended in 20 µL DNase/RNase free water followed by the addition of positive control (Synthetic RNA Control 2) and negative control (1 µg human genome cDNA) (Fig. 1b v). After the RT-LAMP reaction, a clear colour difference was shown between the positive controls and negative controls, indicating a high retention of enzymatic activity after 3 days of dried storage at room temperature (Fig. 1b vi). We found freeze-dried samples were more stable than vacuum-dried samples. Freeze-dried reaction mixes stored at room temperature for 15 days showed as good performance as day 0 mixes, indicating the dried kits can be stored for at least 15 days (Supplementary Fig. S3). The further test shows that the dried kits can be stored at room temperature for at least two months.

### Optimised RT-LAMP reaction mix maintains high sensitivity and specificity

**Figure 2:**
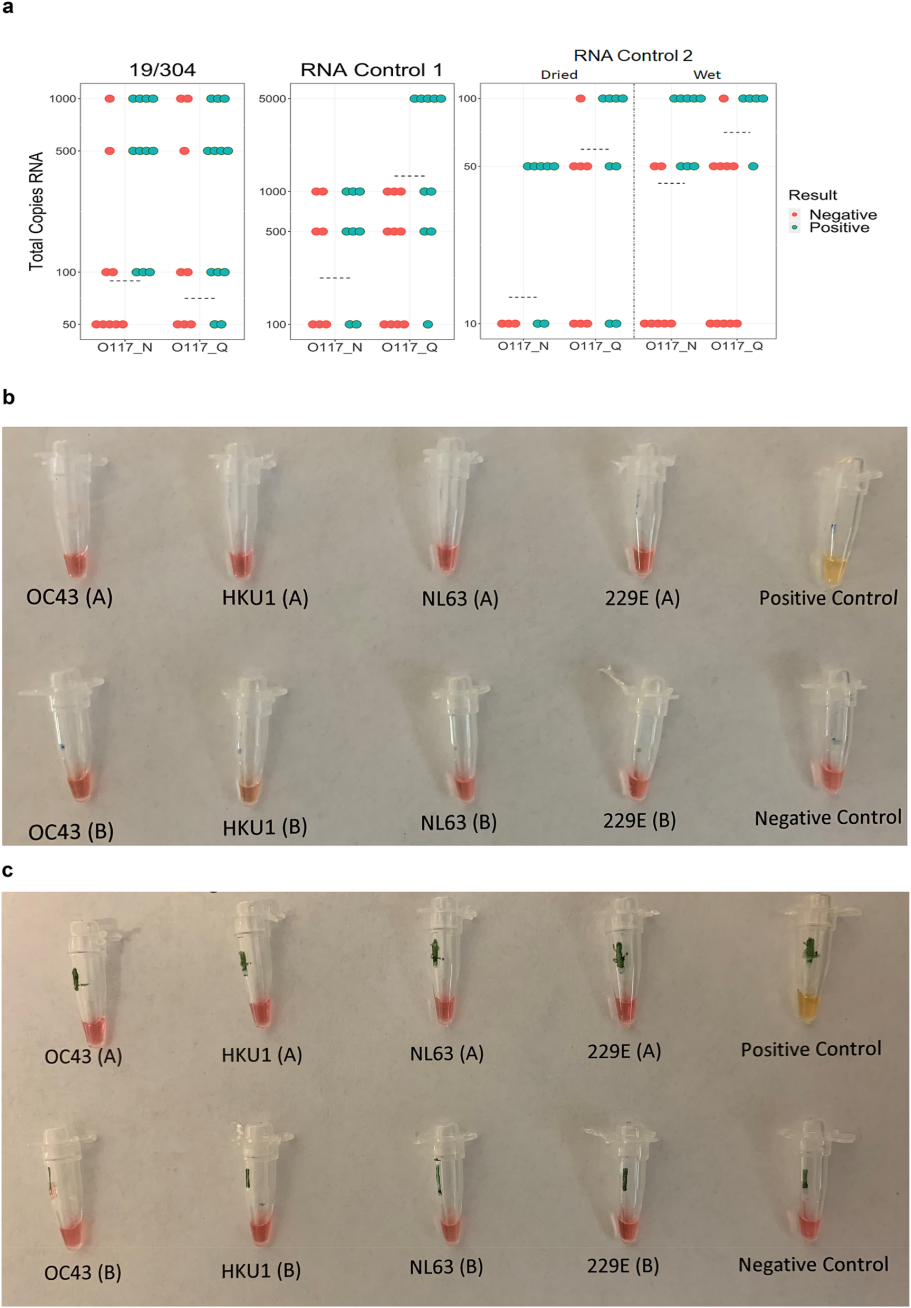
Sensitivity and specificity of improved RT-LAMP assay. (**a**) Determination of 50% endpoints for O117_Q and O117_N preparations of RT-LAMP assay. Full length transcripts were serially diluted in buffer AVE to achieve the total RNA input/reaction indicated on the y axis. Each dot represents one experimental replicate. Dashed lines indicate 50% endpoint as calculated by the Reed-Muench method. Kits were dried except where indicated for RNA Control 2. Full length transcript indicated on figure. Dried reaction mix containing (**b**) O117_N and (**c**) O117_Q were tested against non-SARS human-infective coronaviruses. A and B represent two technical replicates.

The 50% endpoints of detection (50EP) of dried kits of the RT-LAMP assay containing O117_N and O117_Q were determined using three full-length transcripts serially diluted in buffer AVE (Table 2). The RNA templates were research reagent 19/304 (NIBSC, UK), Synthetic RNA control 1—MT007544.1 and Synthetic RNA control 2 MN908947.3 (Twist BioScience, US). The testing results are summarised in Table 2, Fig. 2a and Supplementary Fig. S4. It shows that 50EP against 19/304 were 71 (O117_Q) and 89 (O117_N) copies per reaction (25-μL). The 50EP of Synthetic RNA Control 1 was 131 and 224 copies per reaction for O117_Q and O117_N, respectively. Synthetic RNA Control 2 demonstrated 50EP of 60 and 13 copies per reaction for O117_Q and O117_N, respectively. Additionally, we also tested the 50EP for detection of Synthetic RNA control 2 in wet reaction mixes. The level of detectable RNA for O117_Q and O117_N in wet reaction mix were 71 and 42 copies per reaction. The 50EP for dried reaction mixes containing O117_Q targeting 19/304 and Synthetic RNA Control 2 had a mean of 65 copies per reaction (95% CI 57.3–72.9). The 50EP for dried reaction mixes containing O117_N and targeting 19/304 and Synthetic RNA control 2 had a mean of 51 copies (95% CI − 1.6 to 104.0). These results suggest that the oligo switch in O117 primers has a minimal impact on the LAMP sensitivity for viral RNA detection and the 50EP is 60–131 RNA copies per 25-μL reaction.

**Table 2:** The 50% end-point of detection using full-length transcript standards.

Full-length transcript	Dried kit with O117_Q^a^	Dried kit with O117_N^a^
19/304	71	89
RNA control 1	131	224
RNA control 2	60	13
RNA control 2 (wet kit)	71	42

^a^The total copy number per 25-μL reaction.

The ability of the optimised RT-LAMP assay to discriminate SARS-CoV-2 from human-infective seasonal coronaviruses was assessed using RNA extracted from stored clinical samples previously demonstrated to be infected with respiratory pathogens. The RNA samples from infected patient samples positive for betacoronaviruses (OC43 and HKU1) and alphacoronaviruses (229E and NL63) were used for specificity testing of O117_Q LAMP assay. Neither replicate of the seasonal coronaviruses tested by O117_Q produced a positive result, even using an extended reaction time (40 instead of 30 min) (Fig. 2b,c). This result suggests that O117_Q primer set had a high specificity for SARS-CoV-2 and can discriminate other human-infective coronaviruses.

### Quantitative assessment of colorimetric readout

**Figure 3:**
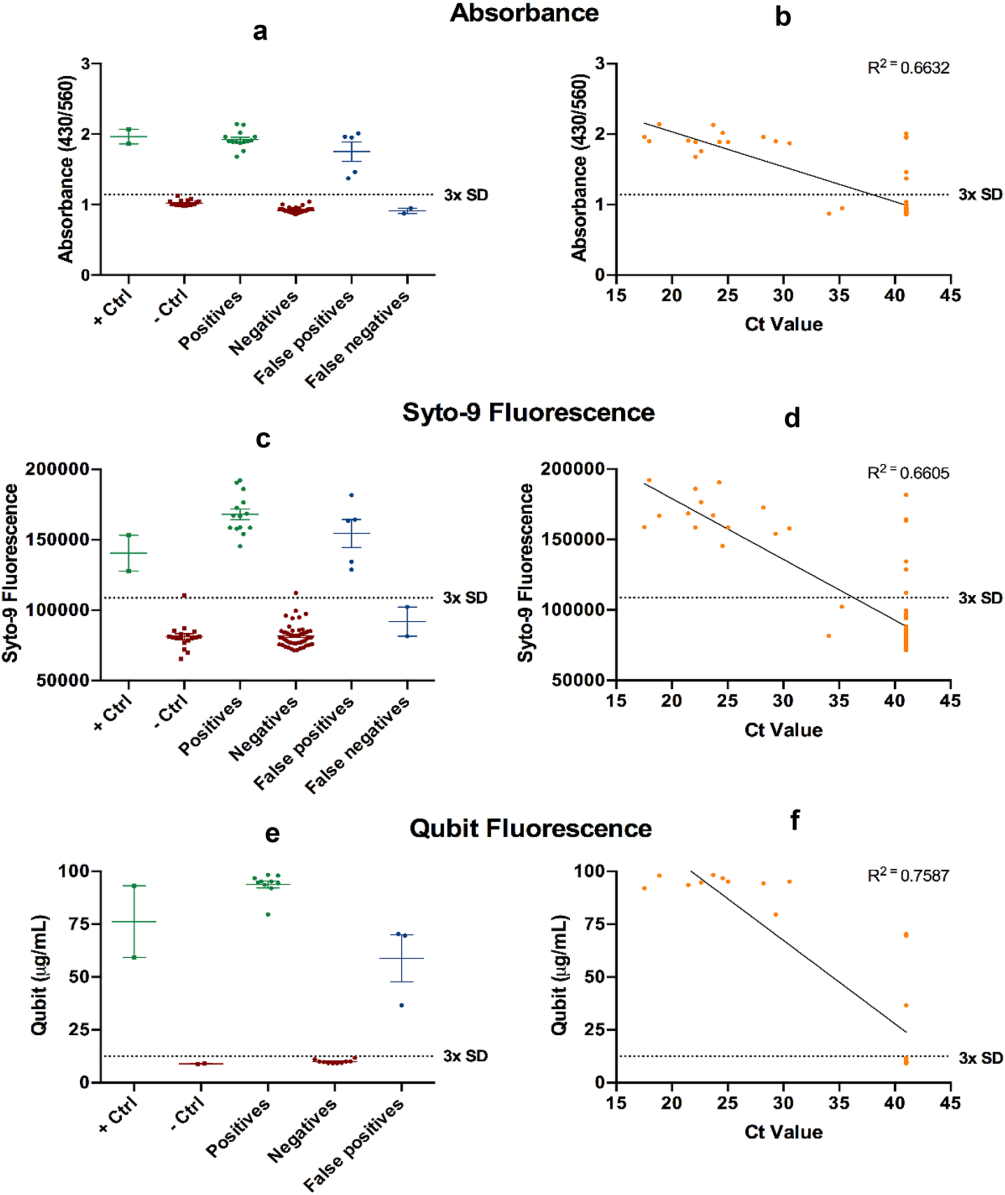
Quantitative evaluation of pH-dependent colorimetric RT-LAMP readout. Three quantitative methods (**a**) Absorbance using 430/560 nm ratio; (**b**) SYTO 9 fluorescence using 485 nm (excitation) and 500 nm (emission); and (**c**) Qubit fluorescence using Qubit 2.0 fluorometer were used to assess the RT-LAMP result. (**d**–**f**) are the correlation analysis between RT-qPCR and the three quantitative evaluations, respectively. Each dot represents one experimental replicate. Dotted lines indicate 3× standard deviations above the negative controls.

Reading the colorimetric RT-LAMP assay has an element of subjectivity. To overcome any ambiguity and potential for inter-user and inter-lab variation when using a visually-assessed colorimetric readout, we sought to establish quantitative measurements for differentiating positive and negative samples (Fig. 3). The first method uses the ratio of the absorbance at 430 nm and 560 nm to quantitatively assess the color change of the 72 swab samples (Supplementary Fig. S5). The result is consistent with the colorimetric readout by the naked eye (Fig. 3a,b). Fluorescent dyes SYTO 9 and Qubit were also used to detect the products of the RT-LAMP reaction. Specifically, RT-LAMP products were stained with SYTO 9 and read using a microplate reader (Fig. 3c,d) or stained with Qubit BR DNA dye and read with a Qubit fluorometer (Fig. 3e,f). Positive samples were defined as having values greater than three times the standard deviation of negative samples (n = 18). As the Qubit is a portable, compact, and gives an accurate readout of fluorescence, we randomly selected a set of ten positive and negative samples as identified previously using RT-qPCR to verify its applicability for POCT. Overall, all three methods demonstrated good agreement with the pH-dependent colorimetric RT-LAMP readout, validating their use as a quantitative measurement to indicate RT-LAMP outcomes. Specifically, absorbance, SYTO9, and Qubit revealed a 2.1, 2.1 and 9.4 fold change between SARS-CoV-2 positive and negative patient samples, respectively. Qubit reagents and fluorometer reading demonstrated a good performance.

### Clinical validation with RNA extract from patient samples

The performance of wet reaction mix containing O117_Q was evaluated using SARS-CoV-2 RNA extracts from 198 nasopharyngeal swab samples. The clinical RNA samples were assessed by both RT-qPCR and RT-LAMP in parallel and the results were shown in Supplementary Table S1. Negative controls and positive controls show the expected results (Table S1 and Supplementary Fig. S5).

**Figure 4:**
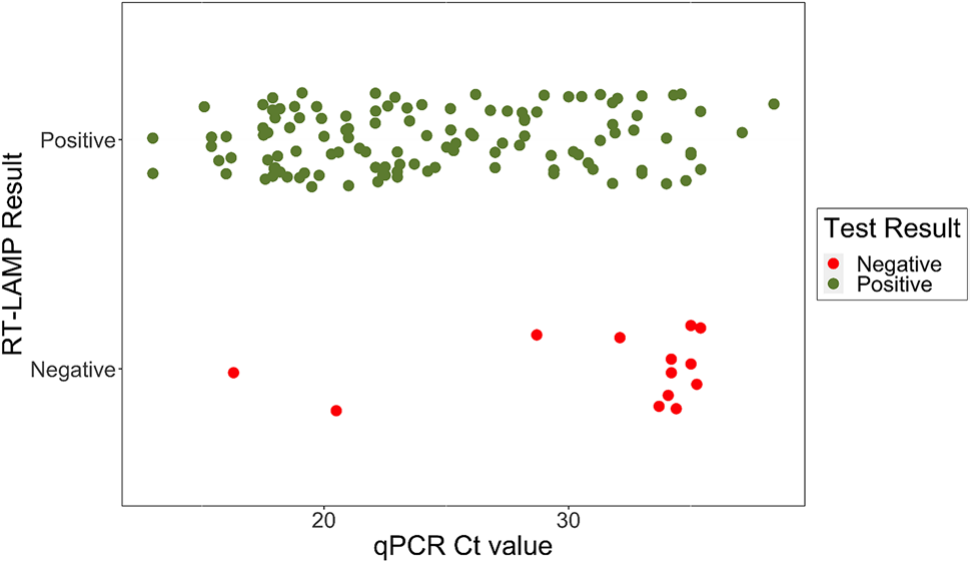
Detection of SARS-CoV-2 from RNA extracts from swab and saliva specimen. Comparison of RT-LAMP for detecting SARS-CoV-2 RNA in extract vs RT-qPCR Ct value. RT-LAMP results were read by colorimetric changes (see Supplementary Fig. S5).

Compared to RT-qPCR, RT-LAMP demonstrated an overall sensitivity of 90.2% (119 detected/132 positive samples; 95% CI 83.8–94.7%) and specificity of 92.4% (61 detected/66 negative samples; 95% CI 83.2–97.5%) (Table 3). There were five false positives detected by RT-LAMP when elution buffer was AVE (Qiagen, UK). After we changed the elution buffer to RNase free water, the false positive has been completely eliminated in RT-LAMP assay (Supplementary Table S1). In total, there were 115 positive samples which Ct values were less than 34, the RT-LAMP assay was able to detect 110 of them with 95.6% sensitivity (Fig. 4 and Supplementary Table S1). The 13 false negative samples may have been due to the degradation of extracted RNA or the low SARS-CoV-2 copy numbers in the samples (Fig. 4).

**Table 3:** Summary of Clinical testing results.

	Clinical RNA testing (Swab and Saliva)
Samples	198 (72 UK and 126 Greece RNA extracts) Positive RT-PCR: 132 (16 UK + 116 Greece RNA extracts) Negative RT-PCR: 66 (56 UK + 10 Greece RNA extracts)
Overall sensitivity (95% CI)	90.2% (83.8–94.7)
Overall specificity (95% CI)	92.4% (83.2–97.5)
Overall percentage agreement (OPA)	91%
Detection time	15–30 min

## Discussion

The rapid spread of SARS-CoV-2, including to resource limited areas, requests the development of novel, rapid methods for detecting infection in individuals. The standard diagnostic tool, RT-qPCR, cannot be readily deployed outside of large diagnostic laboratories due to the necessary technical expertise, sophisticated instrumentation, and costly reagents required for sample preparation and processing. The RT-LAMP assay is an ideal POCT as the only required equipment is a simple heating platform, which could be a heating block, dry incubator, water bath or thermal cycler. The assay is simple to perform, delivering an accurate result rapidly that can be easily interpreted by several different detection systems including fluorescence and absorbance detection, or simple visual inspection in a POC setting. Effective surveillance depends on the frequency of testing and test turnaround time, which has been shown to be only marginally improved by using a test with a high sensitivity^22^.

### A short oligo switch to minimise false positives

We have introduced several adaptations to improve on the performance of our previously reported RT-LAMP assay^11^ that differentiate us from other published RT-LAMP assays for SARS-CoV-2 detection. To minimize the risk of false positive due to unspecific amplification or primer dimers in RT-LAMP^23–25^, we introduce a short oligo switch with sequence complementary to the FIP primer, which ‘locks’ FIP from random self-amplification in the absence of the target RNA. In the presence of the target RNA sequence of SARS-CoV-2, the switch is competitively inhibited for binding the FIP primer and the RT-LAMP reaction proceeds. The addition of the switch decreased the risk of self- or off-target amplification (Fig. 1) and did not demonstrably impair the sensitivity of the assay (Fig. 2a).

### Detection limit of this RT-LAMP assay

We have demonstrated that the RT-LAMP assay is sensitive and highly specific for SARS-CoV-2 RNA. Neither O117_N nor O117_Q primer set produced false positive results over eight reactions of samples containing the four seasonal coronaviruses. The 50% endpoint values for transcripts 19/304 and RNA control 2 are largely in agreement and demonstrate that the RT-LAMP assay can readily detect < 100 copies per reaction of SARS-CoV-2 RNA. The substantially higher 50% endpoint for RNA control 1 may be due to improper initial quantification during shipment or transcript degradation. While a trend towards increased sensitivity in O117_N as one may expect is noted, no statistically significant difference between the O117_N and O117_Q preparations was found when averaged across the 19/304 and RNA Control 2 transcripts, but this may be a consequence of the low sample size.

The RT-LAMP assay demonstrated high concordance with a standard RT-qPCR assay in a laboratory setting. RNA extracts from clinical samples were tested using the RT-LAMP assay and showed reasonably high level of overall percentage agreement (OPA) 91% compared to RT-qPCR.

The five false positives detected by our RT-LAMP assay from the RNA extract samples raise the concerns of self-amplification and carryover contamination repeatedly observed in RT-LAMP^23–25^. We have sought to mitigate these risks through the addition of the switch oligonucleotide which demonstrably increases the stability of the reaction. Through freeze-drying the kit components, we also simplify the process for end users and limit the opportunities for onsite carryover contamination.

RT-LAMP assay detects the vast majority of samples from patients who are likely to be very infectious and this assay could be used for rapid identification of individuals with medium to high viral loads to divert these samples away from overburdened diagnostic laboratories. Some previous data has reported that a threshold of Ct = 33 was threshold for which a patient is no longer considered infectious, as viable virus isolated from patients with a Ct value beyond 33 did not generate a positive culture growth^26,27^. More recent data, which remains to be confirmed, suggests that infectious virus was not recovered at a Ct above 24^28^. Since this RT-LAMP assay was able to get 95.6% sensitivity to Ct < 34, it has a clinical value in practice.

An important consideration in this discussion is that Ct values are not directly comparable and may differ by as much as five cycles when compared directly^29,30^. We also found that two sets of primers including O117_Q and S17^11^ can significantly enhance sensitivity (Supplementary Fig. S6). The switch in O117_Q also prevents false positive results using these two sets of primers. This new design will be applied to future clinical validation.

### Drying mixes enables room temperature transport and storage

Drying the master mix simplifies the process for the end user—all that is required is the addition of water and the patient sample. A further advantage of the dried reagent form is its long-term durability and storage at room temperature. Performance of the dried O117_N and O117_Q kits was not compromised by storage at room temperature for 14 days, although the color of the kits might change to yellow after 5 days due to the change of pH. However, pH and the colour of the kits can be re-adjusted to the original pink colour by adding 2.5 μL of 10 mM KOH. The pH corrected kits still worked as normal in response to water control and full viral RNA transcript (Supplementary Fig. S2). A fluorescence quantitative reading showed that the dry kits responded well to viral RNA after the dry kits were stored at room temperature for at least 14 days (See Supplementary Fig. S3), suggesting that Bst 2.0 DNA polymerase and WarmStart reverse transcriptase in NEB mastermix remain active for a long duration under drying storage condition. The dried kits can be transported without cold chain constraints, reducing transport and storage costs, increasing the resilience of the supply chain and providing access to testing for resource limited settings and POCT outside of standard diagnostic laboratories.

### Colorimetric and fluorescent double display to report RT-LAMP results

Colorimetric RT-LAMP detection methods rely on the qualitative assessment of a pH-dependent colour change of phenol red as an indirect indication of polymerase amplification of the target sequence. As visual interpretation of the experimental results could be subjective, we have established three different quantitative methods to overcome these limitations. The first method exploits the absorbance changes associated with phenol red as a function of pH and the other two methods utilized fluorescent dyes and functions independent of pH. The fluorescent methods are particularly attractive as they function independent to the pH changes and could potentially overcome the problem associated with buffers present in the sample collection media affecting the readouts associated with phenol red. Although RT-LAMP reactions have been conducted in a qPCR machine with real time fluorescence measurement, this necessitates the use of a qPCR machine. Here we show that these readouts can be performed accurately using relatively inexpensive equipment that is readily available to many laboratories. Furthermore, these methods provide additional flexibility to the end users such that the quantification methods can be chosen according to equipment availability and/or throughput requirements. Where higher throughput is required, the RT-LAMP reaction can be conducted in a multi-well format optimised for 96 or 384 well plates using a fluorescent dye such as SYTO9 and read with a microplate reader. On the other hand, the Qubit 2.0 is a relatively inexpensive desktop fluorometer requiring only a power source and can be readily integrated into the testing workflow in a variety of diverse settings, including universities, schools, GP clinics and airports. The additional advantage of Qubit allows electronic transfer of data which could be linked to local and national surveillance programmes.

Thus, given the high accuracy of our LAMP assay, the potential use of multiple detection systems (including portable options such as Qubit), ease of assay performance and the rapid turn around time, we believe this assay could be used as an effective and highly practical first-line screening tool. This RT-LAMP assay shows high accuracy, acceptable sensitivity, and rapid turnaround time, thereby potentially providing a strategic and affordable way to manage surveillance of the SARS-COV-2 public health crisis.

## Supplementary Information

Supplementary Information.
