# Supplementary material for: Clinical validation of optimised RT-LAMP for the diagnosis of SARS-CoV-2 infection
Source: Sci Rep. 2021 Aug 10;11:16193. doi: 10.1038/s41598-021-95607-1 (PMC8355225; doi:10.1038/s41598-021-95607-1)
Supplement: Supplementary file 1 — Supplementary Information. [file 41598_2021_95607_MOESM1_ESM.pdf]

Supplementary information

Clinical Validation of Optimised RT-LAMP for the Diagnosis of SARS-CoV-2 infection

Boon Lim*[1,2], Jeremy Ratcliff*[3], Dorota A. Nawrot*[4], Yejiong Yu[1,2], Harshmeena R. Sanghani[4], Chia-Chen Hsu[1,2], Leon Peto[5], Simon Evans[5], Susanne H Hodgson[5,6], Aikaterini Skeva[7], Maria Adam[7], Maria Panopoulou[7], Christos E Zois[7], Katy Poncin[8], Sridhar R Vasudevan[4], Siqi Dai [1,2], Shuai Ren [1,2], Hong Chang[9], Zhanfeng Cui[1,2,9], Peter Simmonds[3], Wei E. Huang**[1,9] and Monique I. Andersson**[5,10]

[1]*Department of Engineering Science, University of Oxford, Parks Road, OX1 3PJ, Oxford, UK.*

[2]*Institute of Biomedical Engineering, Department of Engineering Science, University of Oxford, Oxford, OX3 7DQ, UK.*

[3]*Peter Medawar Building for Pathogen Research, Nuffield Department of Medicine, University of Oxford, OX1 3SY, Oxford, UK.*

[4]*Department of Pharmacology, University of Oxford, Mansfield Road, Oxford, UK.*

[5]*Department of Microbiology, Oxford University NHS Foundation Trust, Oxford, UK.*

[6]*Jenner Institute, Nuffield Department of Medicine, University of Oxford, Oxford, UK.*

[7]*Democritus University of Thrace, University Hospital of Alexandroupolis, Greece.*

[8]*Sir William Dunn School of Pathology, University of Oxford, Oxford, UK*

[9]*Oxford Suzhou Centre for Advanced Research (OSCAR), China, University of Oxford.*

[10]*Nuffield Division of Clinical Laboratory Science, Radcliffe Department of Medicine, University of Oxford, Oxford, UK*

* These authors contributed equally to this work

** For correspondence, E-mail wei.huang@eng.ox.ac.uk and Monique.Andersson@ouh.nhs.uk

Supplementary information

Supplementary Table S1. Performance of the improved RT-LAMP assay in detecting SARS-CoV-2 from RNA extract isolated from patient samples.

**RT-LAMP, May-June.2020 UK**							
**Sample**	**RT-qPCR Result**	**Ct Value**	**RT-LAMP Result**	**Sample**	**RT-qPCR Result**	**Ct Value**	**RT-LAMP Result**
**B1**	NOT DETECTED	-	NEGATIVE	**E1**	NOT DETECTED	-	NEGATIVE
**B2**	NOT DETECTED	-	NEGATIVE	**E2**	NOT DETECTED	-	NEGATIVE
**B3**	NOT DETECTED	-	NEGATIVE	**E3**	NOT DETECTED	-	NEGATIVE
**B4**	NOT DETECTED	-	NEGATIVE	**E4**	NOT DETECTED	-	NEGATIVE
**B5**	NOT DETECTED	-	NEGATIVE	**E5**	NOT DETECTED	-	NEGATIVE
**B6**	NOT DETECTED	-	NEGATIVE	**E6**	NOT DETECTED	-	POSITIVE (False positive)
**B7**	DETECTED	24.54	POSITIVE	**E7**	NOT DETECTED	-	NEGATIVE
**B8**	NOT DETECTED	-	NEGATIVE	**E8**	NOT DETECTED	-	NEGATIVE
**B9**	DETECTED	21.45	POSITIVE	**E9**	DETECTED	28.2	POSITIVE
**B10**	DETECTED	29.29	POSITIVE	**E10**	DETECTED	34.07	NEGATIVE (False negative)
**B11**	DETECTED	25.02	POSITIVE	**E11**	NOT DETECTED	-	NEGATIVE

**B12**	NOT DETECTED	-	NEGATIVE	**E12**	NOT DETECTED	-	NEGATIVE
**C1**	NOT DETECTED	-	NEGATIVE	**F1**	NOT DETECTED	-	NEGATIVE
**C2**	NOT DETECTED	-	NEGATIVE	**F2**	NOT DETECTED	-	NEGATIVE
**C3**	NOT DETECTED	-	NEGATIVE	**F3**	NOT DETECTED	-	NEGATIVE
**C4**	NOT DETECTED	-	NEGATIVE	**F4**	NOT DETECTED	-	NEGATIVE
**C5**	NOT DETECTED	-	NEGATIVE	**F5**	NOT DETECTED	-	NEGATIVE
**C6**	NOT DETECTED	-	NEGATIVE	**F6**	NOT DETECTED	-	NEGATIVE
**C7**	NOT DETECTED	-	NEGATIVE	**F7**	NOT DETECTED	-	NEGATIVE
**C8**	NOT DETECTED	-	NEGATIVE	**F8**	DETECTED	22.11	POSITIVE
**C9**	DETECTED	23.69	POSITIVE	**F9**	DETECTED	35.24	NEGATIVE (False negative)
**C10**	DETECTED	17.53	POSITIVE	**F10**	DETECTED	17.96	POSITIVE
**C11**	DETECTED	30.54	POSITIVE	**F11**	NOT DETECTED	-	NEGATIVE
**C12**	NOT DETECTED	-	NEGATIVE	**F12**	NOT DETECTED	-	NEGATIVE
**D1**	NOT DETECTED	-	NEGATIVE	**G1**	NOT DETECTED	-	POSITIVE (False positive)
**D2**	NOT DETECTED	-	NEGATIVE	**G2**	NOT DETECTED	-	NEGATIVE
**D3**	NOT DETECTED	-	NEGATIVE	**G3**	NOT DETECTED	-	NEGATIVE

**D4**	NOT DETECTED	-	POSITIVE (False positive)	**G4**	NOT DETECTED	-	NEGATIVE
**D5**	NOT DETECTED	-	POSITIVE (False positive)	**G5**	NOT DETECTED	-	NEGATIVE
**D6**	NOT DETECTED	-	NEGATIVE	**G6**	NOT DETECTED	-	NEGATIVE
**D7**	NOT DETECTED	-	NEGATIVE	**G7**	NOT DETECTED	-	NEGATIVE
**D8**	NOT DETECTED	-	POSITIVE (False positive)	**G8**	NOT DETECTED	-	NEGATIVE
**D9**	DETECTED	18.86	POSITIVE	**G9**	DETECTED	24.24	POSITIVE
**D10**	DETECTED	22.61	POSITIVE	**G10**	DETECTED	22.11	POSITIVE
**D11**	NOT DETECTED	-	NEGATIVE	**G11**	NOT DETECTED	-	NEGATIVE
**D12**	NOT DETECTED	-	NEGATIVE	**G12**	NOT DETECTED	-	NEGATIVE

RT-LAMP, May-June 2021, Greece							
**Sample**	**RT-qPCR Result**	**Ct Value**	**RT-LAMP Result**	**Sample**	**RT-qPCR Result**	**Ct Value**	**RT-LAMP Result**
57942	DETECTED	15.1	Positive	58999	NOT DETECTED	-	NEGATIVE
57813	DETECTED	17.5	Positive	58998	NOT DETECTED	-	NEGATIVE
58030	DETECTED	17.9	Positive	58982	NOT DETECTED	-	NEGATIVE
58348	DETECTED	22.1	Positive	58979	NOT DETECTED	-	NEGATIVE
57981	DETECTED	22.5	Positive	58978	NOT DETECTED	-	NEGATIVE
58405	DETECTED	26.8	Positive	58977	NOT DETECTED	-	NEGATIVE
58071	DETECTED	27	Positive	58976	NOT DETECTED	-	NEGATIVE
58063	DETECTED	28.1	Positive	58975	NOT DETECTED	-	NEGATIVE
58620	DETECTED	19.5	Positive	60009	NOT DETECTED	-	NEGATIVE
58349	DETECTED	22.1	Positive	60010	NOT DETECTED	-	NEGATIVE
58285	DETECTED	25.17	Positive	53323	DETECTED	16.2	Positive
58036	DETECTED	31.3	Positive	53443	DETECTED	17.7	Positive
58017	DETECTED	34.2	NEGATIVE (False negative)	54216	DETECTED	31.8	Positive

58356	DETECTED	34.4	NEGATIVE (False negative)	59295	DETECTED	33.7	NEGATIVE (False negative)
57991	DETECTED	35	NEGATIVE (False negative)	53004	DETECTED	15.4	Positive
58038	DETECTED	35.4	NEGATIVE (False negative)	53002	DETECTED	16	Positive
58800	DETECTED	13	Positive	52977	DETECTED	19	Positive
58801	DETECTED	15.7	Positive	53003	DETECTED	19.8	Positive
58799	DETECTED	17.9	Positive	53834	DETECTED	21	Positive
58802	DETECTED	20.9	Positive	53056	DETECTED	25.18	Positive
58811	DETECTED	34.2	NEGATIVE (False negative)	53148	DETECTED	26	Positive
58789	DETECTED	18.6	Positive	53140	DETECTED	34	Positive
57981	DETECTED	22.5	Positive	52831	DETECTED	18	Positive
58951	DETECTED	23.1	Positive	52835	DETECTED	18	Positive
53505	DETECTED	25.4	Positive	52829	DETECTED	18.8	Positive
58405	DETECTED	26.2	Positive	52833	DETECTED	19	Positive
58320	DETECTED	28.7	NEGATIVE (False negative)	52832	DETECTED	20	Positive

53579	DETECTED	34.6	Positive	52876	DETECTED	20.3	Positive
53516	DETECTED	35	Positive	52830	DETECTED	21	Positive
54471	DETECTED	18.1	Positive	53124	DETECTED	22.4	Positive
54256	DETECTED	19.1	Positive	52566	DETECTED	13	Positive
54512	DETECTED	19.2	Positive	52824	DETECTED	17.7	Positive
54172	DETECTED	22.2	Positive	52567	DETECTED	18.2	Positive
54472	DETECTED	28.2	Positive	52825	DETECTED	20.5	NEGATIVE (False negative)
54254	DETECTED	29.4	Positive	52826	DETECTED	27.5	Positive
54473	DETECTED	31.3	Positive	52828	DETECTED	29.4	Positive
54217	DETECTED	17.5	Positive	52823	DETECTED	30	Positive
49321	DETECTED	18.2	Positive	52803	DETECTED	33	Positive
58755	DETECTED	24.2	Positive	52549	DETECTED	16	Positive
58680	DETECTED	25.3	Positive	52542	DETECTED	18.5	Positive
54195	DETECTED	28	Positive	52407	DETECTED	23	Positive

54475	DETECTED	28.7	Positive	52408	DETECTED	23	Positive
54025	DETECTED	29	Positive	52410	DETECTED	24	Positive
53201	DETECTED	32.67	Positive	52406	DETECTED	27	Positive
53622	DETECTED	16.3	NEGATIVE (False negative)	52548	DETECTED	28.2	Positive
53359	DETECTED	17.9	Positive	52409	DETECTED	35	Positive
53988	DETECTED	19.7	Positive	52179	DETECTED	20.59	Positive
54016	DETECTED	19.9	Positive	52007	DETECTED	23	Positive
53978	DETECTED	20.9	Positive	59322	DETECTED	31.8	Positive
53629	DETECTED	26.1	Positive	59920	DETECTED	32.8	Positive
53625	DETECTED	30.8	Positive	59642	DETECTED	34	Positive
53826	DETECTED	31.9	Positive	52037	DETECTED	34.3	Positive
53744	DETECTED	17.6	Positive	52019	DETECTED	35.4	Positive
53569	DETECTED	21.7	Positive	52031	DETECTED	35.4	Positive
53823	DETECTED	23.5	Positive	52029	DETECTED	15.4	Positive

53787	DETECTED	30.2	Positive	52182	DETECTED	21	Positive
53762	DETECTED	30.4	Positive	52178	DETECTED	22.9	Positive
53512	DETECTED	33	Positive	59823	DETECTED	27.3	Positive
53977	DETECTED	34.8	Positive	52180	DETECTED	31	Positive
53767	DETECTED	35	NEGATIVE (False negative)	59324	DETECTED	31.8	Positive
52381	DETECTED	23.4	Positive	52354	DETECTED	32	Positive
60197	DETECTED	32.1	NEGATIVE (False negative)	59528	DETECTED	33	Positive
51951	DETECTED	37.1	Positive	52009	DETECTED	38.4	Positive

(a)

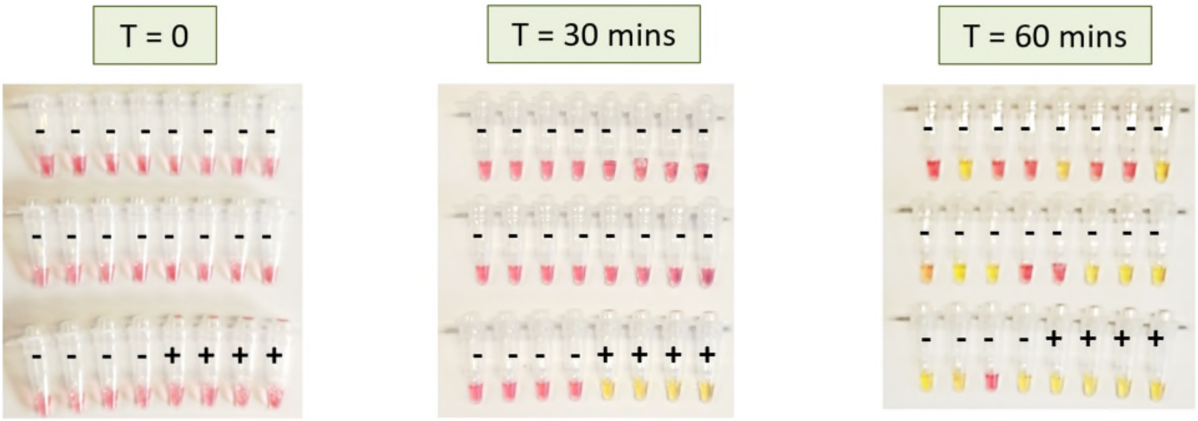

(b)

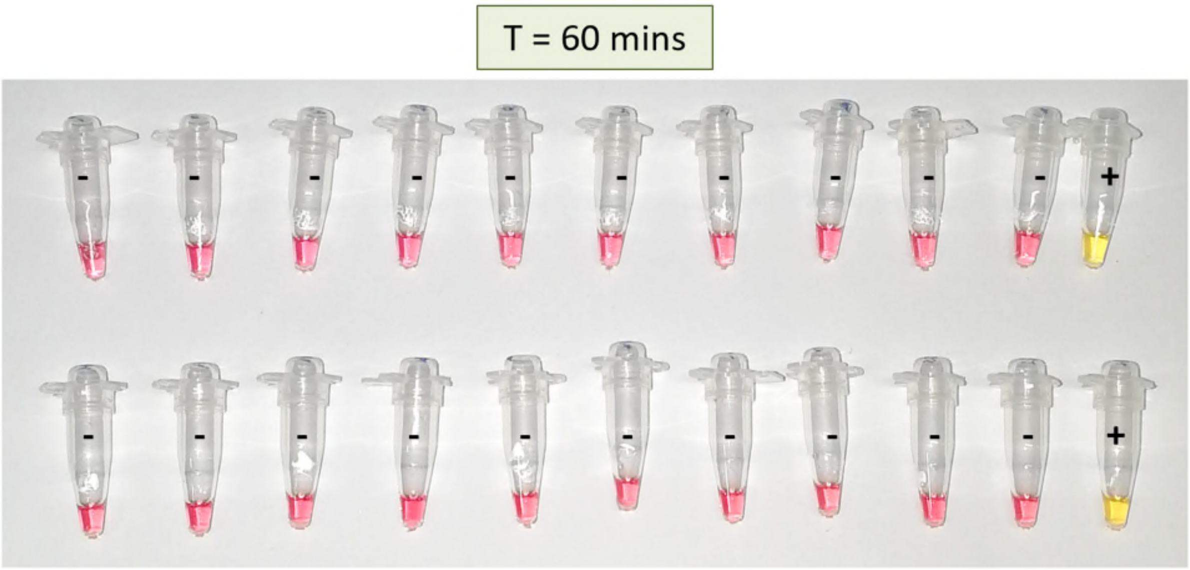

Supplementary Figure S1. The switch can stabilise RT-LAMP performance within an hour of incubation. O117_N generated 60% false positive results (12/20 reactions) due to self- and off-target amplification in human RNA control sample after incubating at 65 ^{o}C for 60 minutes. O117_Q still shows negative to human RNA control as long as 60 minutes at 65 ^{o}C reaction.

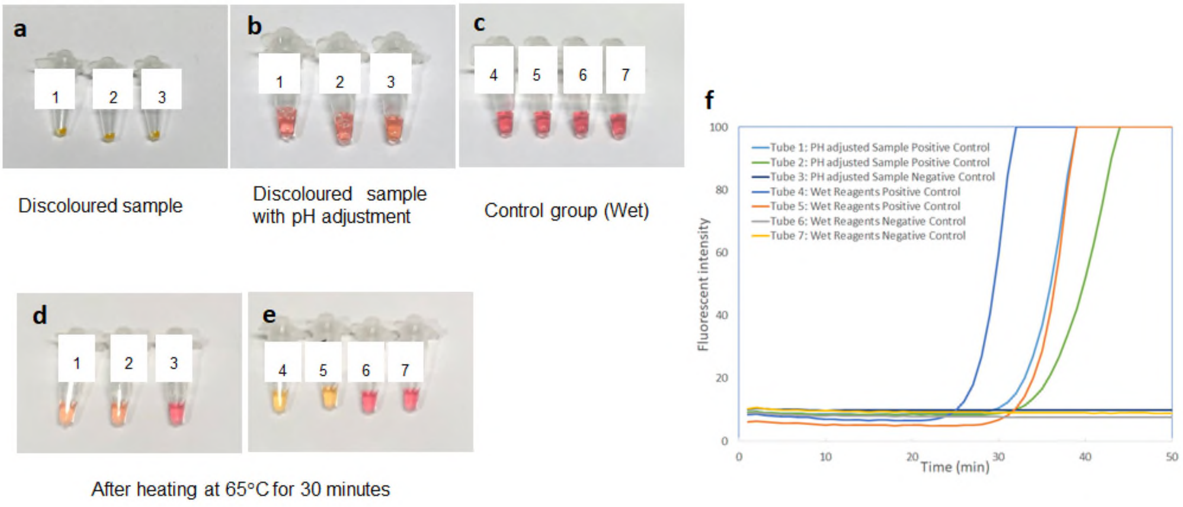

Supplementary Figure S2. RT-LAMP performance of pH corrected decolored kits that were stored at room temperature for 7 days. Colour change of dried to yellow after storage of 7 days (**a**). The pH of kits can be adjusted by adding KOH and the colour of the adjusted mixes (**b)** produced a similar colour to original fresh mixes (**c**). The pH colour-corrected mixes can perform colorimetric detection of Synthetic RNA Control 2 - MN908947.3 (Twist Bioscience) at 200 copies in final reaction solution (**d** and **e**). 1 and 2: pH adjusted mixes with viral RNA, 3. Blank control of pH adjusted mix with water. 4 and 5: control mixes with viral RNA. 6 and 7: Blank control of fresh mixes with water. (**f**) Fluorescent detection of the amplification process of pH corrected and fresh mixes.

Day 0 in room temperature (20 ºC)

Before reaction T= 0 min

	Before reconstitution	After reconstitution
**Batch 1**	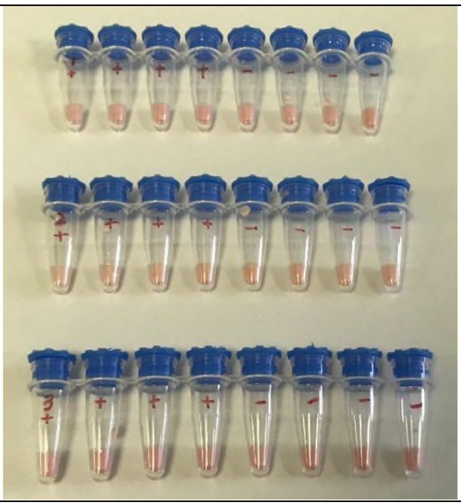	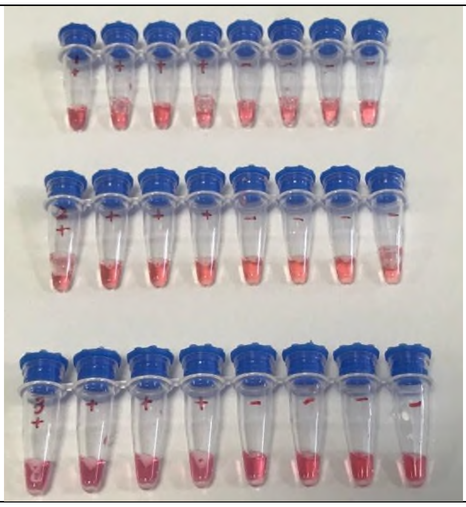
**Batch 2**		
**Batch 3**		
	1 2 3 4 5 6 7 8	1 2 3 4 5 6 7 8

Day 0

After reaction T= 30 min

	Before 65°C for 30 minutes	After 65°C for 30 minutes
**Batch 1**	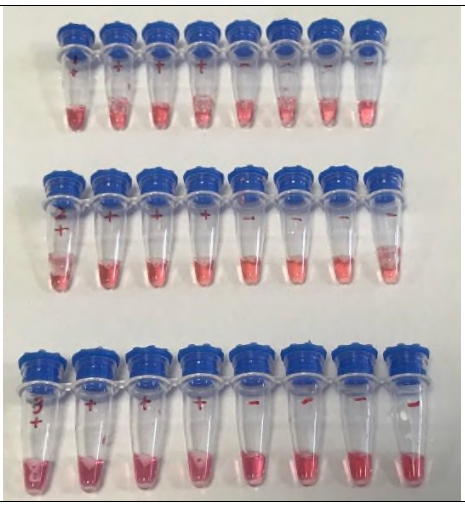	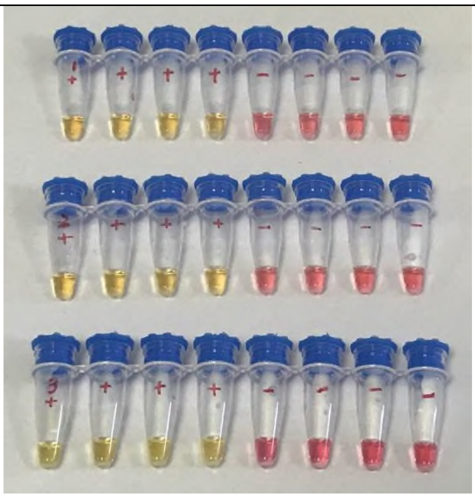
**Batch 2**		
**Batch 3**		
	1 2 3 4 5 6 7 8	1 2 3 4 5 6 7 8

Day 15 in room temperature (20 ºC)

Before reaction T= 0 min

<table>
<tr><th></th><th>Before reconstitution</th><th>After reconstitution</th></tr>
<tr><td>Batch 1</td><td rowspan="3">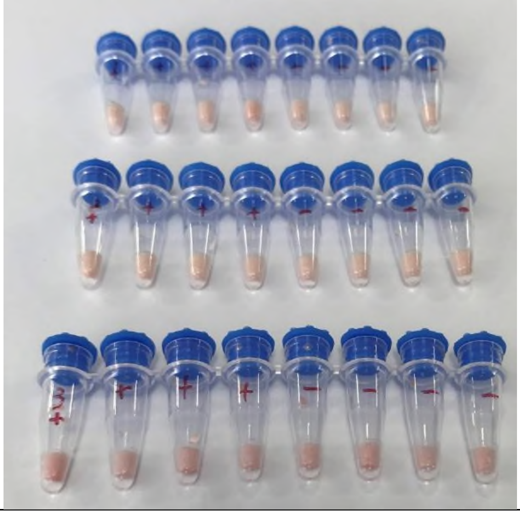</td><td rowspan="3">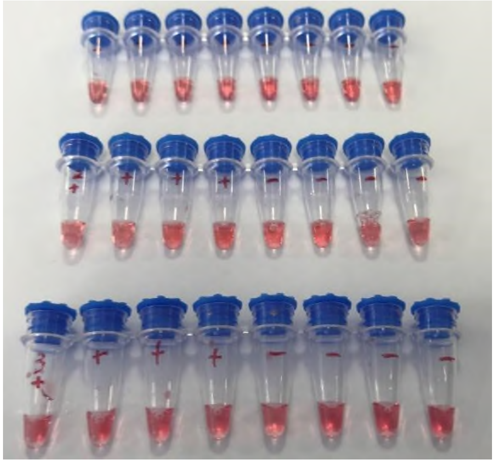</td></tr>
<tr><td>Batch 2</td></tr>
<tr><td>Batch 3</td></tr>
<tr><td></td><td>1 2 3 4 5 6 7 8</td><td>1 2 3 4 5 6 7 8</td></tr>
</table>

Day 15

After reaction T= 30 min

<table>
<tr><th></th><th>Before 65°C for 30 minutes</th><th>After 65°C for 30 minutes</th></tr>
<tr><td>Batch 1</td><td rowspan="3">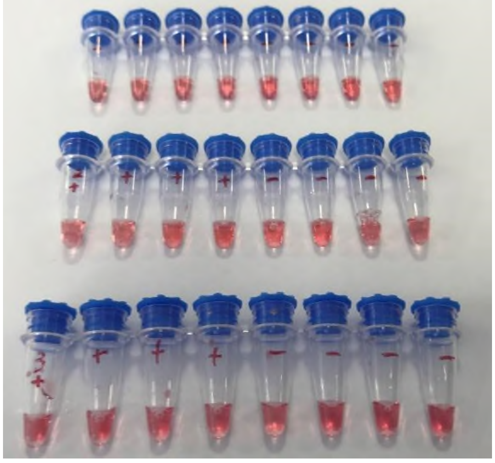</td><td rowspan="3">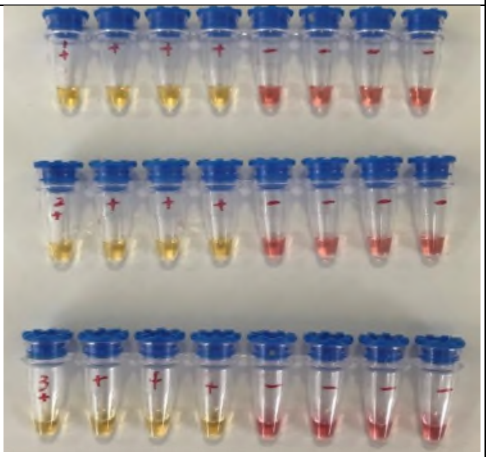</td></tr>
<tr><td>Batch 2</td></tr>
<tr><td>Batch 3</td></tr>
<tr><td></td><td>1 2 3 4 5 6 7 8</td><td>1 2 3 4 5 6 7 8</td></tr>
</table>

Supplementary Figure S3. Long-term storage of optimized RT-LAMP dried reaction mixes at room temperature. Dried mixes were stored for day 0 (A) and day 15 (B) at room temperature. Dried mixes were reconstituted with 20 µL DNase/RNase free water followed by the addition of 5 µL of Synthetic RNA Control 2 - MN908947.3 (40 copies/µL, Twist Bioscience) or human genome DNA as negative control. (iii) Tubes were incubated at 65 °C for 30 minutes. Each time three batches and each batch three replicates were carried out.

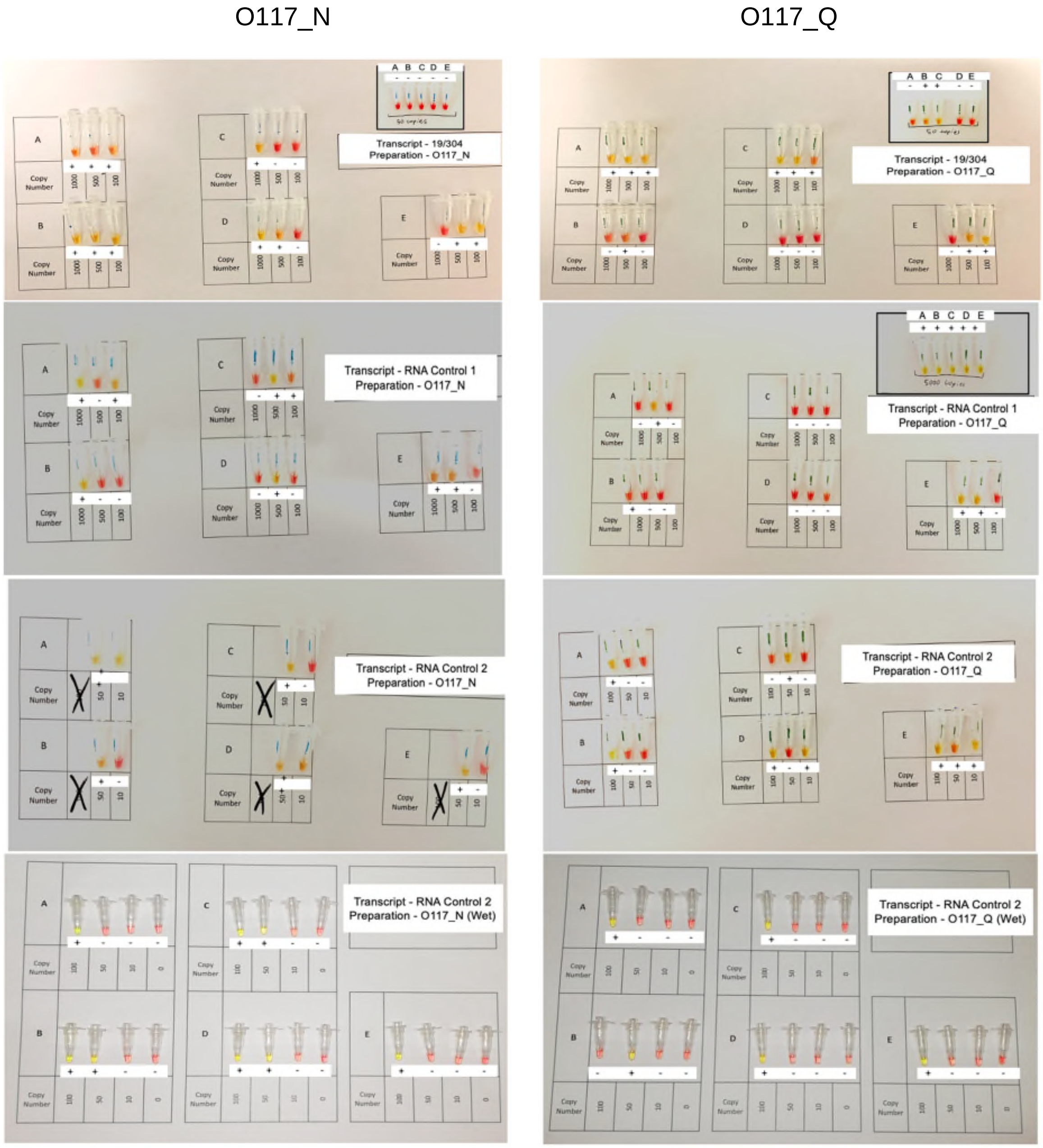

Supplementary Figure S4. Sensitivity analysis of improved RT-LAMP assay. SARS-CoV-2 RNA transcript and reaction mix used in each experiment are labelled on the top right white box of each image. '+' indicates a positive result while '-' indicates a negative result. A, B, C, D, E represents five technical replicates.

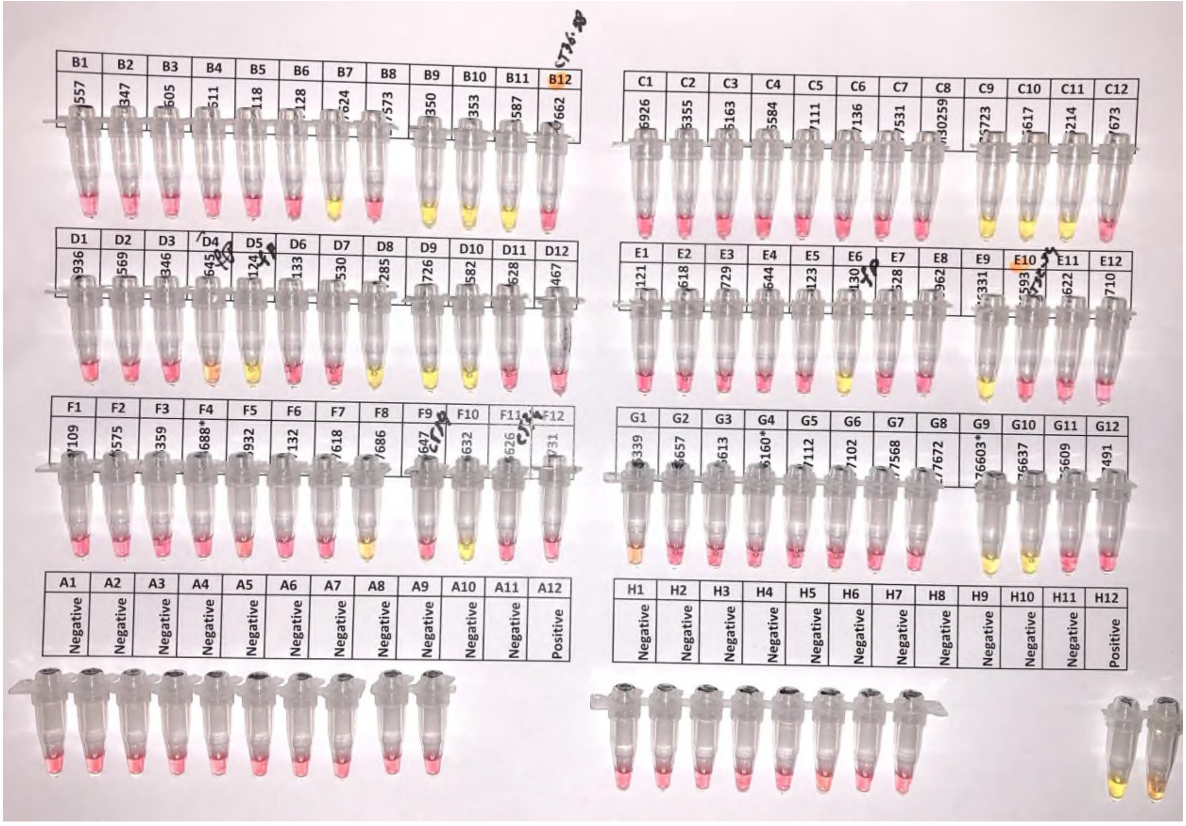

Supplementary Figure S5a. Detection of SARS-CoV-2 from clinical RNA extracts by RT-LAMP in John Radcliff hospital Oxford. Performance of RT-LAMP assay using wet reaction mix containing O117_Q on detecting SARS-CoV-2 from clinical RNA extract samples. Data was collected on 22nd May 2020. A1-A10 andH1-H8 are the negative controls, H11 and H12 are the positive controls.

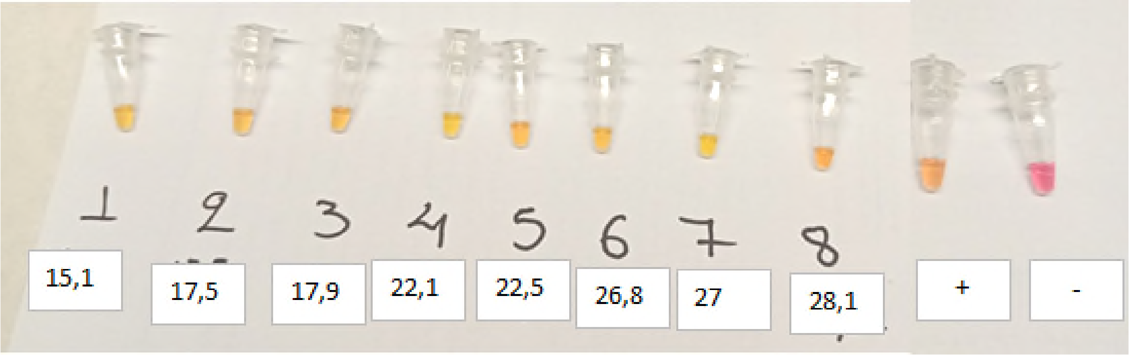
1
2
3
4
5
6
7
8
15,1
17,5
17,9
22,1
22,5
26,8
27
28,1
+
-

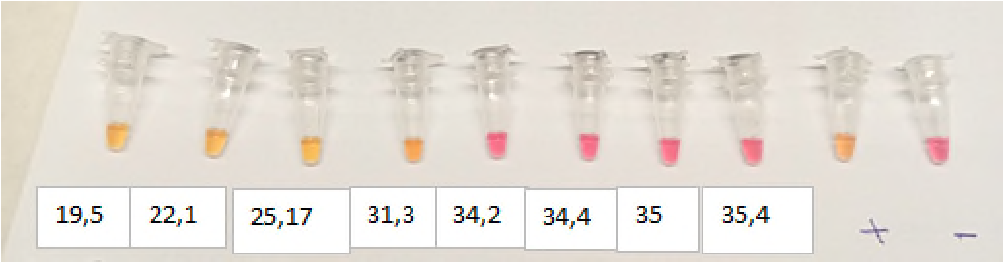
19,5
22,1
25,17
31,3
34,2
34,4
35
35,4
+
-

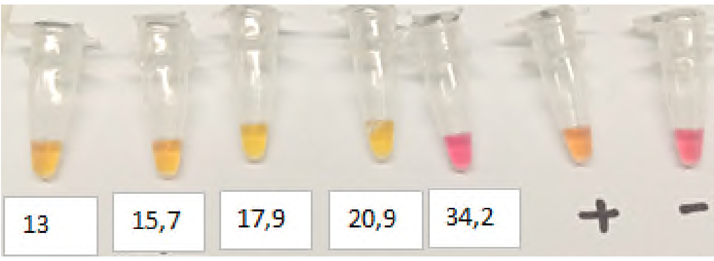
13
15,7
17,9
20,9
34,2
+
-

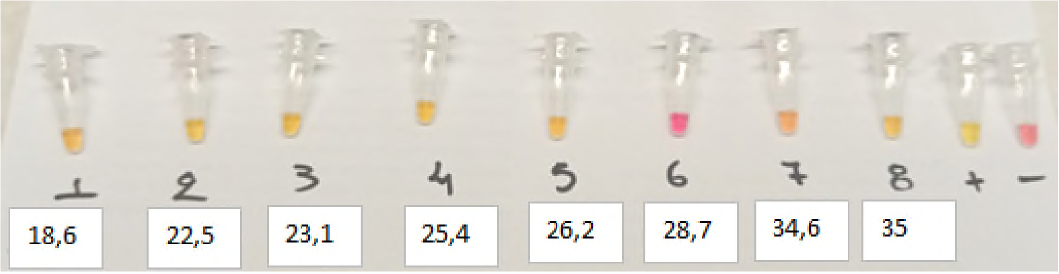
1
2
3
4
5
6
7
8
+
-
18,6
22,5
23,1
25,4
26,2
28,7
34,6
35

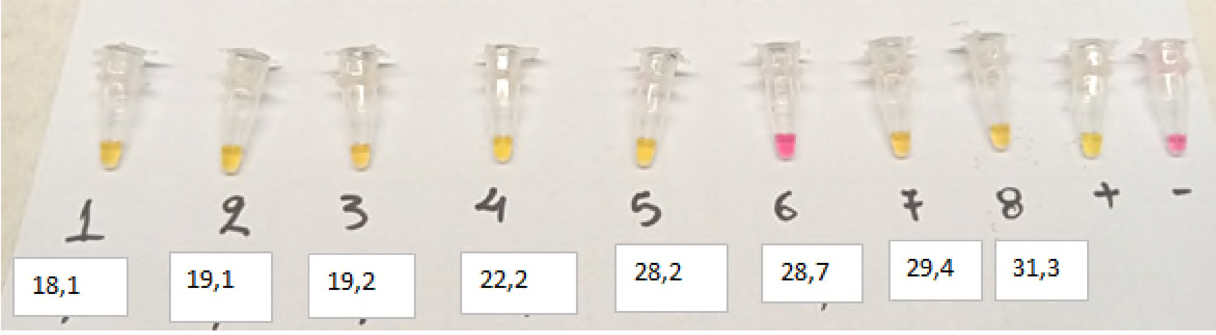
1
2
3
4
5
6
7
8
+
-
18,1
19,1
19,2
22,2
28,2
28,7
29,4
31,3

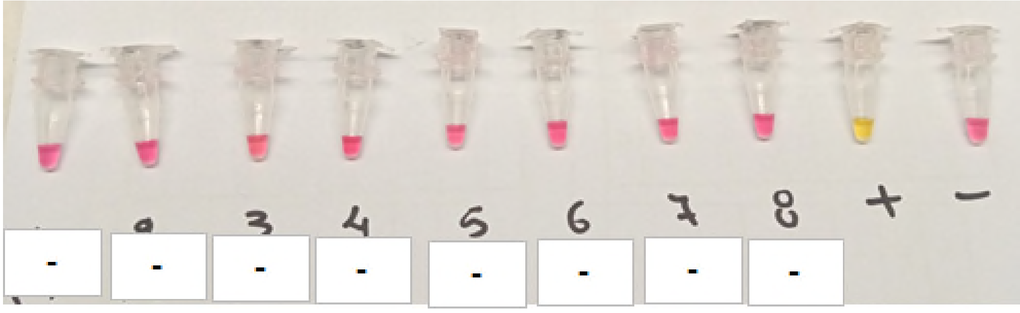
3
4
5
6
7
8
+
-
-
-
-
-
-
-
-
-

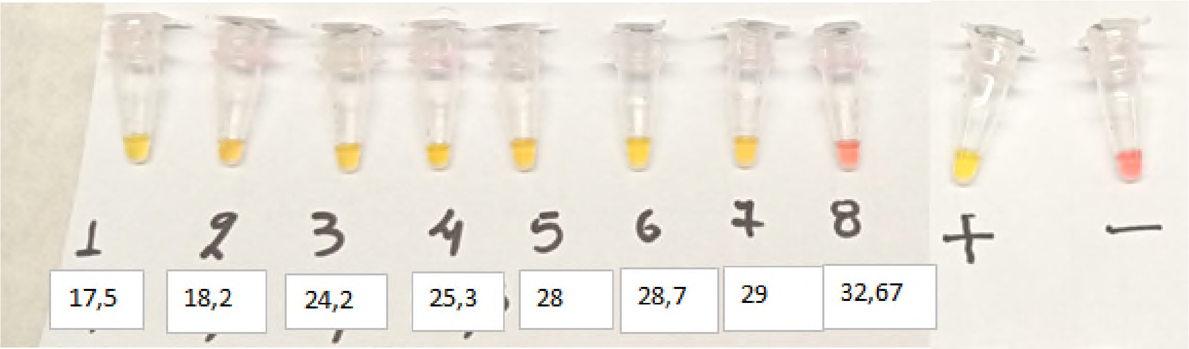
1
2
3
4
5
6
7
8
+
-
17,5
18,2
24,2
25,3
28
28,7
29
32,67

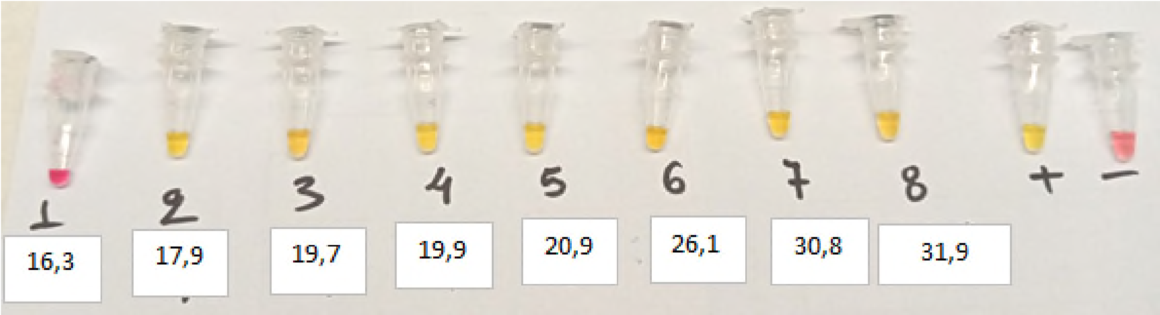
1
2
3
4
5
6
7
8
+
-
16,3
17,9
19,7
19,9
20,9
26,1
30,8
31,9

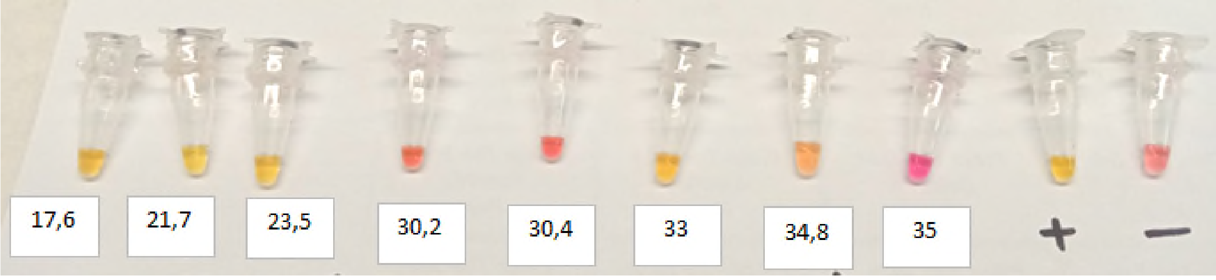
17,6
21,7
23,5
30,2
30,4
33
34,8
35
+
−

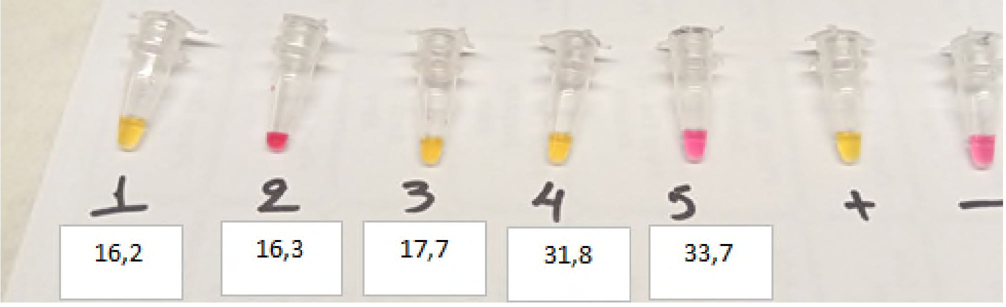
1
2
3
4
5
+
−
16,2
16,3
17,7
31,8
33,7

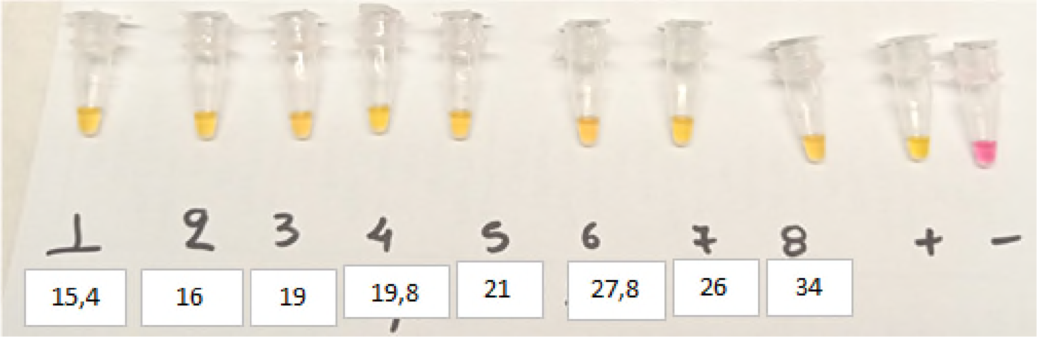
1
2
3
4
5
6
7
8
+
−
15,4
16
19
19,8
21
27,8
26
34

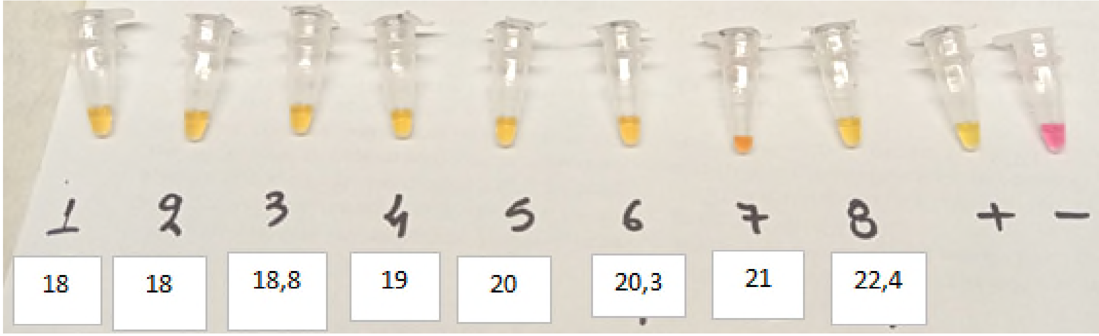
1
2
3
4
5
6
7
8
+
−
18
18
18,8
19
20
20,3
21
22,4

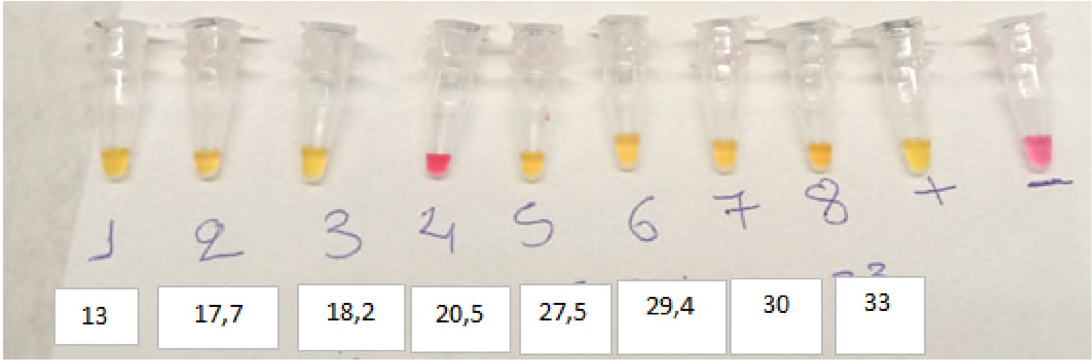
1
2
3
4
5
6
7
8
+
—
13
17,7
18,2
20,5
27,5
29,4
30
33

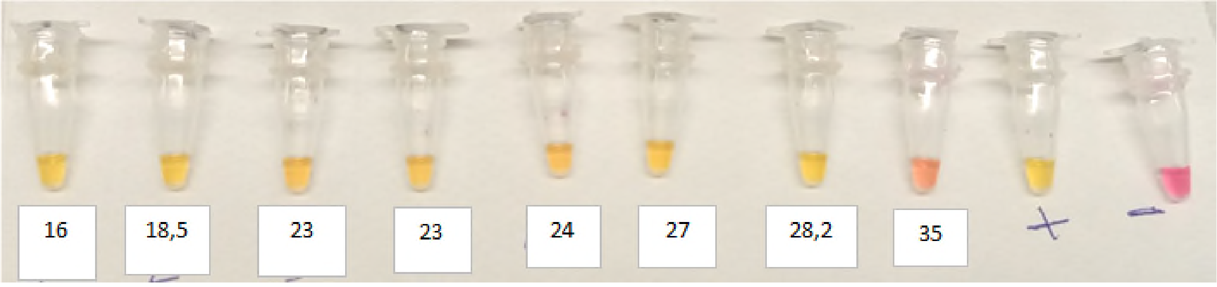
16
18,5
23
23
24
27
28,2
35
+
-

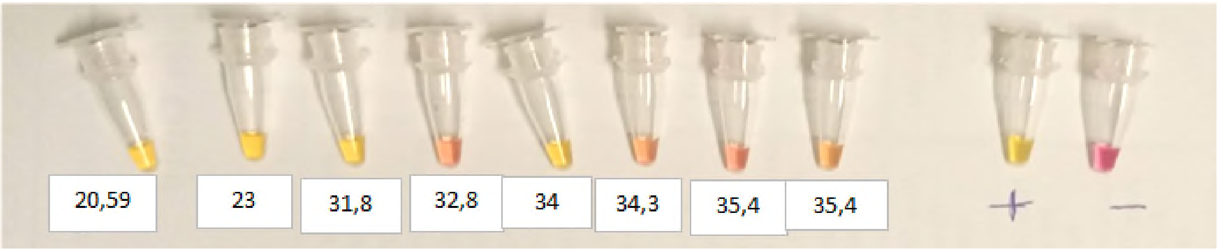
20,59
23
31,8
32,8
34
34,3
35,4
35,4
+
—

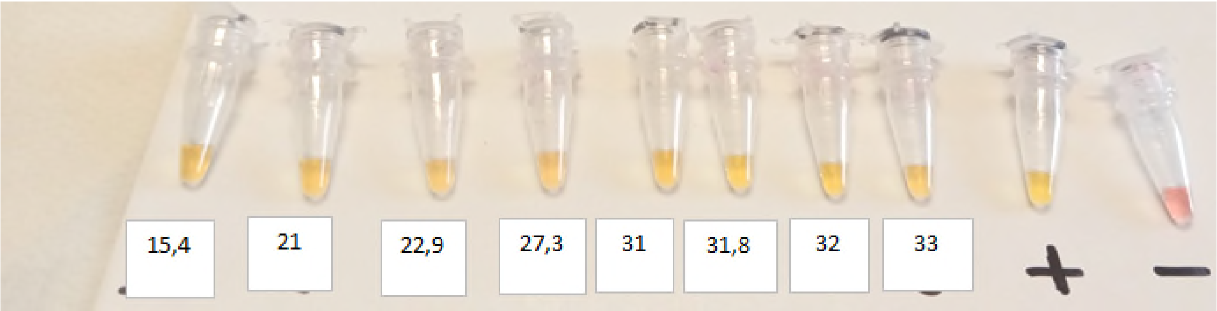
15,4
21
22,9
27,3
31
31,8
32
33
+
—

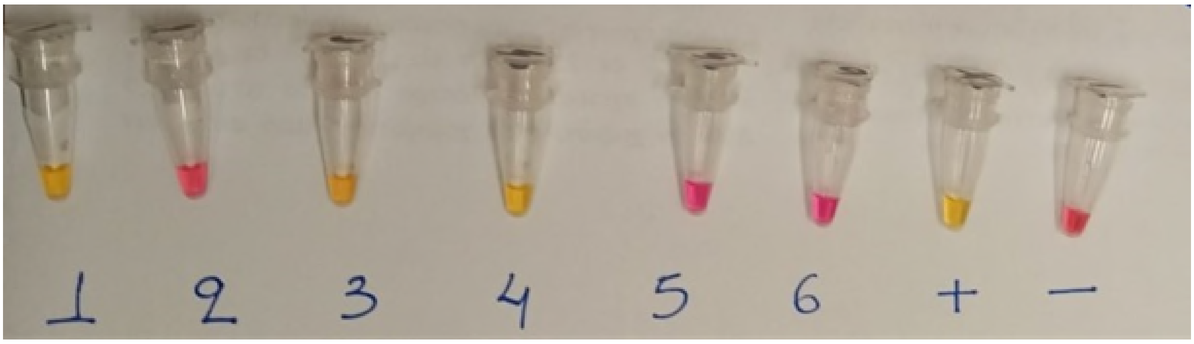

Supplementary Figure S5b. Detection of SARS-CoV-2 from 126 RNA extracts (116 positives and 10 negatives) by RT-LAMP in University Hospital of Alexandroupolis, Greece. Performance of RT-LAMP assay using freeze drying reaction mix containing O117_Q on detecting SARS-CoV-2 from RNA extract from saliva samples. PC: positive control and NC: negative control.

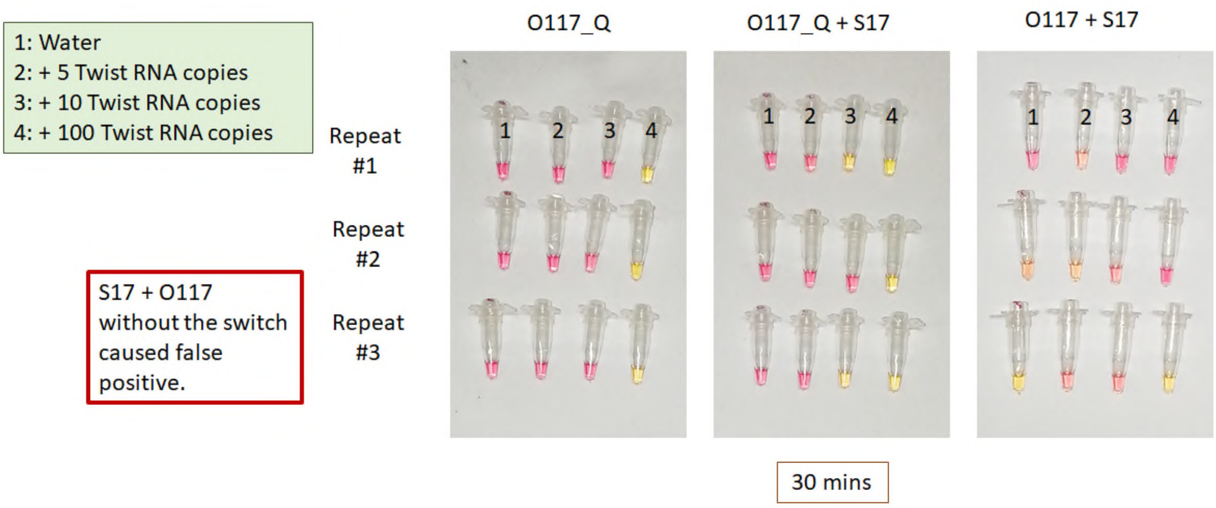

Supplementary Figure S6. Two sets of primers (O117_Q and S17) in one reaction enhanced detection sensitivity of SARS-CoV-2 virus in RT-LAMP assay. Two sets of primers with the switch showed enhanced sensitivity and no false postiive results, whilst those without the switch resulted in false positive results.
